# SWI/SNF-Deficient Sinonasal Carcinomas: A Retrospective Case Series of 17 Patients from a Single Institution

**DOI:** 10.3390/jcm15082939

**Published:** 2026-04-13

**Authors:** Zijun Qiu, Aodeng Surita, Xiaowei Wang, Yingxian Qian, Zhenzhen Zhu, Wei Lv

**Affiliations:** 1 Department of Otolaryngology–Head and Neck Surgery, Peking Union Medical College Hospital, Chinese Academy of Medical Sciences and Peking Union Medical College, No. 1, Shuaifuyuan, Wangfujing, Dongcheng District, Beijing 100730, China; qzjhjqw1109@163.com (Z.Q.); aodengsurita@163.com (A.S.); 13701397599@163.com (X.W.); 2 Department of Pathology, Peking Union Medical College Hospital, Chinese Academy of Medical Sciences and Peking Union Medical College, No. 1, Shuaifuyuan, Wangfujing, Dongcheng District, Beijing 100730, China; qyx17302592015@163.com

**Keywords:** SWI/SNF complex, SMARCB1/INI1, SMARCA4/BRG1, sinonasal carcinoma, teratocarcinosarcoma, diagnosis, multimodality treatment

## Abstract

**Objectives**: We aimed to characterize the clinicopathologic features, treatment, and outcomes of three types of Switch/Sucrose Nonfermentable (SWI/SNF)-deficient sinonasal carcinomas (SDSCs), thereby expanding the spectrum of these rare entities and facilitating early diagnosis. **Methods**: We designed a retrospective single-center case series to analyze the clinicopathological features of 17 patients with SMARCB1-deficient sinonasal carcinoma (*n* = 10), SMARCA4-deficient carcinoma (*n* = 6) and SMARCA4-deficient sinonasal teratocarcinosarcoma (TCS) (*n* = 1) treated between 2018 and 2025, and reviewed the relevant literature. **Results**: The cohort included 14 males and 3 females, aged 26 to 69 years (mean, 47 years). SMARCB1-deficient sinonasal carcinomas predominantly involved the ethmoid sinus (6 of 8 patients), presenting epistaxis (7 of 10 patients), nasal obstruction (5 of 10 patients), and ocular symptoms (4 of 10 patients). SMARCA4-deficient sinonasal carcinomas mainly arose in the nasal cavity (3 of 4 patients), characterized by nasal obstruction (4 of 6 patients), and epistaxis or purulent rhinorrhea (4 of 6 patients); ocular symptoms were less common (2 of 6 patients). The TCS patient had left nasal cavity and ethmoid involvement with nasal obstruction and purulent rhinorrhea. Most patients presented with advanced-stage disease (T4a, *n* = 9), with skull base (*n* = 6), and orbital (*n* = 3) involvement. Histologically, immunohistochemical analysis confirmed complete SMARCB1 or SMARCA4 loss (complete in carcinomas and partial in TCS), diffuse CK positivity, and high Ki-67 indices. Treatment modalities included: chemotherapy and immunotherapy without surgery (*n* = 2), radical surgery with adjuvant chemoradiotherapy and immunotherapy (*n* = 2), radical surgery with chemoradiotherapy (*n* = 9), postoperative radiotherapy alone (*n* = 3), and non-radical surgery with chemoradiotherapy (*n* = 1). At a median follow-up of 19 months (range, 8–57 months), 2 patients were lost to follow-up, 3 died, 2 had persistent disease, and 10 remained disease-free. **Conclusions**: SDSC is an aggressive tumor with male predominance and advanced-stage presentation. Early recognition and appropriate immunohistochemical evaluation are essential for timely diagnosis and management. Prospective studies of novel targeted and immunotherapeutic strategies are warranted.

## 1. Introduction

The Switch/Sucrose Nonfermentable (SWI/SNF) complex is a tumor-suppressive chromatin-remodeling complex. Inactivation of different subunits can lead to the development of highly aggressive tumors of varying types and anatomical locations [1]. Given the distinct functional roles of its core subunits, loss of different components may result in divergent patterns of chromatin dysregulation and tumor biology. The complex comprises multiple core subunits (SMARCB1) [2] and two catalytic subunits, SMARCA2 (BRM) and SMARCA4 (BRG1) [1]. Tumors occurring in the nasal cavity and paranasal sinuses are exclusively associated with deficiencies of the SMARCB1 and SMARCA4 subunits. These alterations define four main entities: SMARCB1-deficient carcinoma, SMARCB1-deficient adenocarcinoma, SMARCA4-deficient carcinoma, and SMARCA4-deficient sinonasal teratocarcinosarcoma (TCS) [3]. Currently available immunohistochemical antibodies enable relatively accurate pathological diagnosis of these tumors, which have been incorporated into the latest (5th edition) World Health Organization Classification of Head and Neck Tumors and are recognized as aggressive neoplasms with poor prognosis [4], suggesting that early diagnosis and prompt treatment may improve clinical outcomes [5,6]. Despite increasing recognition, SMARCB1-deficient carcinoma remains rare, and current knowledge is largely based on small case series or isolated reports. Fewer than 200 cases have been reported in earlier studies, although the number of reported cases has increased in recent years. SMARCA4-deficient carcinoma is even rarer, and fewer than 50 cases have been reported in total [7,8,9,10,11]. In this study, we retrospectively analyzed 17 patients with three types of SWI/SNF-deficient Sinonasal Carcinomas (SDSC) including SMARCB1-deficient sinonasal carcinoma, SMARCA4-deficient sinonasal carcinoma and SMARCA4-deficient sinonasal TCS, treated at a tertiary medical center over 7 years. SMARCB1-deficient adenocarcinoma was not included due to the absence of eligible cases. Most existing studies on SWI/SNF-deficient sinonasal carcinomas are based on Western populations, while data from Asian cohorts remain limited. Although several case reports and small series from China and other East Asian populations have been described [12,13,14,15], they remain limited, and potential differences in genetic background, clinical presentation, and treatment patterns have not been well characterized. To our knowledge, this represents one of the largest Asian cohorts to date, encompassing multiple histological subtypes and further expanding the disease spectrum of these rare entities. The primary objectives were to delineate the clinical, radiological, and pathological profiles, as well as therapeutic management and outcomes, thereby enhancing awareness among otolaryngologists and pathologists, and potentially facilitating timely diagnosis and intervention.

## 2. Materials and Methods

Patients diagnosed with SDSC at Peking Union Medical College Hospital between 2018 and 2025 were reviewed retrospectively. Patients were included if they had a confirmed histopathological diagnosis of SDSC. This included patients who underwent surgery at our institution, as well as those treated elsewhere who were subsequently referred to our center for pathological consultation or adjuvant therapy. Patients were excluded if the diagnosis was uncertain or if essential pathological data were unavailable. Cases with incomplete follow-up data were included in the analysis based on available information. All pathological diagnoses were independently reviewed and confirmed by at least two experienced pathologists at our institution, and discrepancies were resolved by consensus. For patients referred from external institutions, all available pathological materials were re-evaluated at our center to ensure diagnostic consistency. Immunohistochemical staining was performed using anti-SMARCB1 antibody (clone SMARCB1/3984, Biorbyt, Beijing, China; dilution 1:100) and anti-SMARCA4 antibody (bs-42429R, Bioss, Beijing, China; dilution 1:200). Complete loss was defined as absence of nuclear staining in tumor cells with preserved staining in internal controls, whereas partial loss was defined as decreased or heterogeneous staining. We collected demographic, clinical, radiological, pathological, and immunohistochemical data from medical records. Follow-up data were collected between 1 September and 8 September 2025, through structured telephone interviews. Information collected included clinical symptoms, date of diagnosis, tumor location, surgical history, treatment details, survival status, and the occurrence of metastasis. In addition, detailed information on surgical approaches was not consistently available. Despite efforts to obtain these data through medical records and follow-up, a substantial proportion of cases lacked sufficient detail. Therefore, the surgical approach was not included in the analysis to avoid bias from incomplete data. This study was approved by the Ethics Committee of Peking Union Medical College Hospital (approval number: I-25PJ1351) and verbal informed consent was obtained from all patients involved in the study. Tumor staging was based on the 9th edition of the American Joint Committee on Cancer TNM staging system. Kaplan–Meier survival analysis was performed using SPSS software (Version 27.0, IBM Corp., Armonk, NY, USA) to estimate disease-free survival (DFS), with patients censored at the last follow-up if no event occurred. Due to the limited sample size, no formal comparative statistical tests were performed. Missing data (variables recorded as “NA” due to incomplete medical records, unavailable imaging data, or loss to follow-up) were handled using a complete-case approach. No imputation was performed, and analyses were conducted based on available data for each variable. Treatment strategies were determined based on available clinical records. Radical surgery was defined as complete tumor resection with negative surgical margins (R0 resection). Non-radical surgery was defined as resection with microscopic (R1) or macroscopic (R2) residual disease, or cases in which complete resection was not feasible due to extensive local invasion. However, owing to the retrospective design and the inclusion of patients initially treated at external institutions, detailed information regarding the rationale for treatment selection was not consistently available.

## 3. Results

### 3.1. Clinical Features

This study included 17 patients (14 males, 3 females; male proportion 82.4%) with a median age of 47 years (range, 25–69 years). Due to missing or incomplete data for certain variables (tumor location or staging), denominators may vary across analyses, and percentages are reported based on available cases for each variable. Information on occupational exposures and prior sinonasal conditions (such as nasal polyps or chronic sinusitis) was not systematically available due to the retrospective design of the study. All patients were from a single-center Chinese cohort, resulting in a relatively homogeneous ethnic background.

- For SMARCB1-deficient carcinomas (*n* = 10), tumor location could not be determined in 2 patients due to unavailable surgical records or imaging data. Among the remaining 8 patients, the ethmoid sinus was the most commonly involved site (*n* = 6). Other sites included the anterior skull base (*n* = 3), maxillary sinus (*n* = 2), and orbital contents (*n* = 2). The most common presenting symptoms were epistaxis (*n* = 7) and nasal obstruction (*n* = 5). Ocular symptoms were observed in 4 patients, while headache, hyposmia, and facial pain were less frequent.
- For SMARCA4-deficient carcinomas (*n* = 6), tumor location data were available for 4 patients only. Among these patients, the nasal cavity (*n* = 3) and anterior skull base (*n* = 3) were the most frequently involved sites, while involvement of orbital contents was noted in 1 patient. Clinically, the most common symptoms were nasal obstruction (*n* = 4) and epistaxis or purulent rhinorrhea (*n* = 4). Ocular symptoms were less frequent (*n* = 2). Headache and facial pain were not reported in this subgroup.
- The SMARCA4-deficient TCS patient (Case 17) had a tumor involving the left nasal cavity and ethmoid sinus, presenting with nasal obstruction and purulent rhinorrhea.

Case 4 had a complex diagnostic history. The patient was initially diagnosed with multiple intracranial anaplastic meningiomas (WHO Grade III) at an external institution and underwent two resections for local recurrence within one year. One and a half years later, the patient presented to our center with maxillary sinus SDSC, which prompted re-evaluation of the previous specimens. Pathological consultation at our hospital confirmed homology between the previous intracranial tumors and the current SDSC. After multidisciplinary discussion, the initial meningiomas were considered likely to represent metastatic SDSC. Another case (Case 14) had concurrent primary hepatocellular carcinoma. None of the other patients had evidence of lymph node involvement or distant metastasis at initial presentation. Detailed clinicopathological features and the specific sites of metastasis for each patient are summarized in Table 1. Among patients who developed distant metastases, the most common sites included the lung, bone, brain, liver, and lymph nodes.

### 3.2. Radiologic and Pathologic Findings

**Figure 1:**
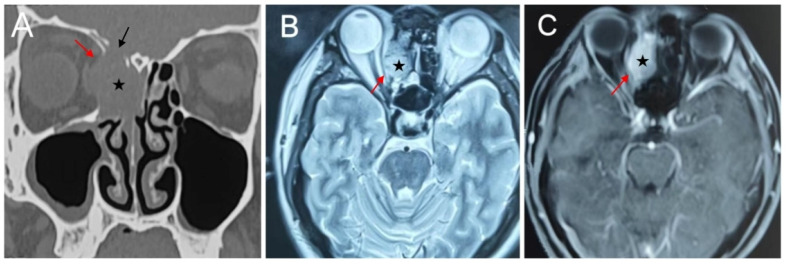
Imaging features of SMARCB1-deficient sinonasal carcinoma (Case 6). (**A**) Coronal noncontrast CT showing a tumor in the right ethmoid sinus (black star), extending through the lamina papyracea into the right orbit (red arrow) and eroding the ethmoidal roof (black arrow). (**B**) Axial T2-weighted MRI demonstrating mildly hyperintense signal in the right ethmoid sinus (black star). (**C**) Post-contrast T1-weighted MRI showing heterogeneous enhancement. The lesion compresses the medial rectus muscle (red arrow) and is closely related to the anterior skull base.

**Figure 2:**
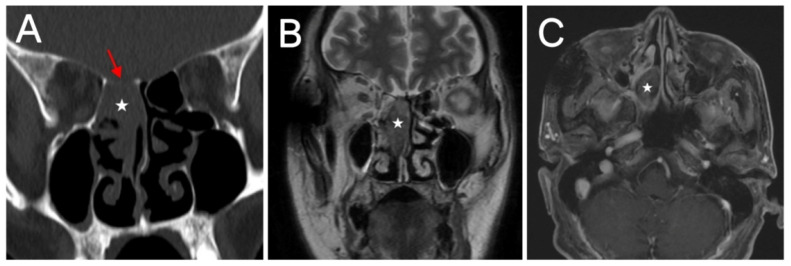
Imaging features of SMARCA4-deficient sinonasal carcinoma (Case 14). (**A**) Coronal noncontrast CT showing a soft tissue mass in the posterior right nasal cavity (white star), poorly demarcated from the anterior skull base (red arrow). (**B**) Coronal T2-weighted MRI showing mildly hyperintense signal. (**C**) Axial post-contrast T1-weighted MRI demonstrating heterogeneous enhancement with indistinct margins relative to adjacent structures.

Preoperative evaluation typically utilizes computed tomography (CT) and magnetic resonance imaging (MRI) to assess lesion extent and guide surgical planning. Radiologically, the exact tumor location could not be accurately confirmed in some cases due to incomplete medical records. Of the 13 patients with detailed staging information, most presented at an advanced stage. Nine (69.2%) had T4a tumors, including 5 SMARCB1-deficient carcinomas and 4 with SMARCA4-deficient carcinomas. These tumors frequently showed extensive infiltration of the maxilla and adjacent nasal bony structures. Orbital invasion was present in 3 patients. In several additional cases, the tumor abutted the periorbita and displaced the orbital contents without definite invasion. Skull base invasion occurred in 6 patients. CT typically demonstrated destruction of the anterior skull base, whereas MRI was useful for evaluating intracranial extension. On contrast-enhanced imaging, these tumors typically demonstrated heterogeneous enhancement of varying degrees (Figure 1 and Figure 2).

**Figure 3:**
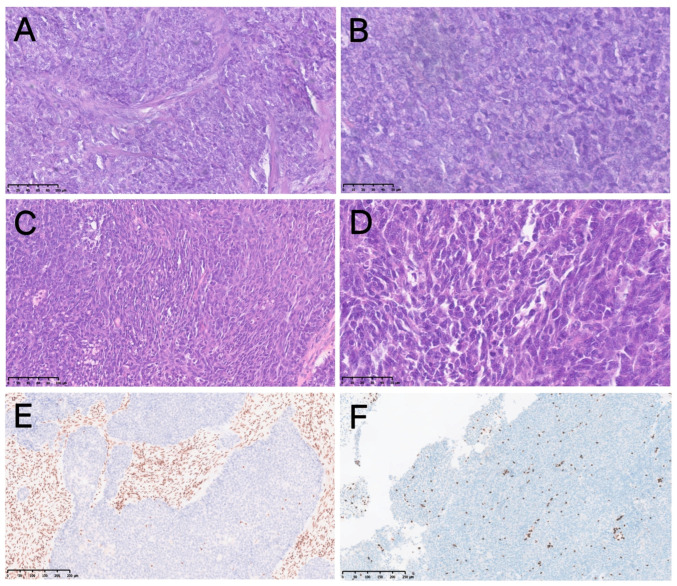
Histopathological features of SMARCB1-deficient and SMARCA4-deficient sinonasal carcinomas. (**A**) SMARCB1-deficient carcinoma showing basaloid morphology with small round blue cells (HE, ×200). (**B**) Higher magnification demonstrating vesicular nuclei and prominent nucleoli (HE, ×400). (**C**) SMARCA4-deficient carcinoma showing solid nests with focal necrosis (HE, ×200). (**D**) Epithelioid morphology with high mitotic activity (HE, ×400). (**E**) Complete loss of SMARCB1 expression. (**F**) Complete loss of SMARCA4 expression.

Histologically, these tumors commonly showed cellular monotony, brisk mitotic activity, and variable necrosis. The tumor cells typically had relatively monomorphic small-to-intermediate round nuclei, without definitive squamous or glandular differentiation (Figure 3A,C). Nonspecific vesicular nuclei can be seen in some cases (Figure 3B,D). The histological appearance of SDSC often overlaps with that of other poorly differentiated or small round blue cell tumors. Accurate diagnosis is challenging without immunohistochemistry (IHC). The pathognomonic feature is loss of SMARCB1 or SMARCA4 expression (Figure 3E,F). In our cohort, all 10 SMARCB1-deficient cases showed complete SMARCB1 loss, with 100% CK positivity, low neuroendocrine marker expression, and Ki-67 indices ranging from 1% to 70%. All six SMARCA4-deficient cases showed complete loss of SMARCA4, diffuse CK positivity, high expression of neuroendocrine markers (CgA/Syn), Ki-67 indices ranging from 70% to 90%, and negative staining for P40/P63. The single SMARCA4-deficient TCS case showed partial loss of SMARCA4, positivity for AE1/AE3 and Vimentin, and a Ki-67 index of 70%. Ki-67 data were unavailable in some cases due to incomplete pathological records from external institutions or limited tissue samples. Given the descriptive nature of this study and the small sample size, missing Ki-67 data were not expected to substantially affect the overall interpretation.

### 3.3. Treatment and Follow-Up

Due to the consultation-based nature of these cases, comprehensive treatment and follow-up data were limited. Follow-up ranged from 8 to 57 months. Of the 13 patients available for follow-up, 3 with SMARCB1-deficient carcinoma died of disease, 1 with SMARCB1-deficient and 1 with SMARCA4-deficient carcinoma were alive with disease at last follow-up. The remaining patients had no evidence of recurrence or distant metastasis.

**Figure 4:**
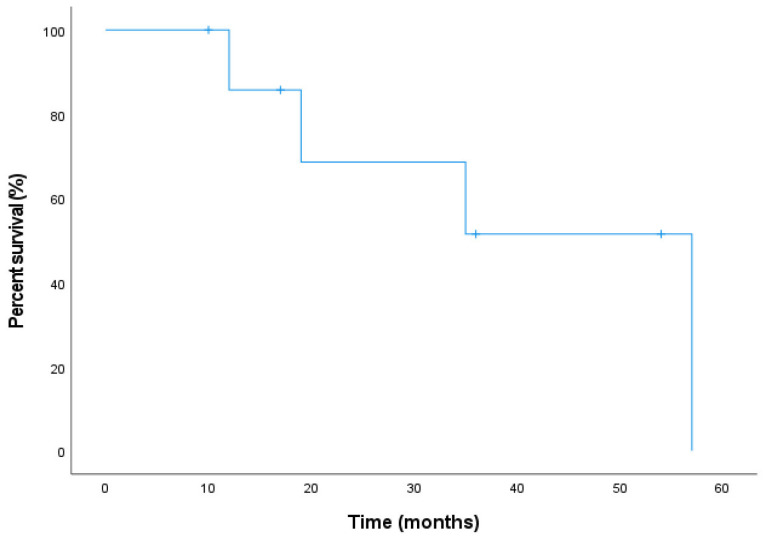
Kaplan–Meier curve of disease-free survival (DFS) in 8 patients with SMARCB1-deficient sinonasal carcinoma available for follow-up.

- For SMARCB1-deficient carcinomas (*n* = 8), two patients (Cases 5 and 9) received chemotherapy and immunotherapy (paclitaxel and nimotuzumab) after biopsy, with no significant tumor response and subsequent disease progression. Salvage surgery and adjuvant therapy were then performed; Case 9 was lost to follow-up, and Case 5 died of obstructive shock due to mediastinal metastasis. One patient (Case 7) underwent non-radical resection due to skull base invasion, received one cycle of chemotherapy (discontinued due to severe adverse effects), followed by 33 fractions of radiotherapy; no progression was observed at 1-year follow-up. The remaining 7 patients underwent radical tumor resection (defined as complete tumor resection with negative surgical margins) as initial treatment; those with skull base invasion required concurrent dural repair and reconstruction. Six patients received postoperative chemoradiotherapy. Among them, Case 10 received additional immunotherapy (pembrolizumab switched to sintilimab) but developed liver metastasis at 1 year postoperatively; Case 1 developed brain metastasis at 41 months, received salvage immunotherapy, targeted therapy, and laser interstitial thermotherapy (LITT), and died at 57 months. Notably, Case 6 received only 30 fractions (60 Gy) of proton therapy (PT) postoperatively but remained disease-free at 17-month follow-up. Among 8 SMARCB1-deficient patients available for follow-up, Kaplan–Meier analysis revealed 1- and 3-year DFS rates of 85.7% and 51.4%, respectively (Figure 4). However, these estimates should be interpreted with caution due to the small sample size and high censoring rate.
- For SMARCA4-deficient carcinomas (*n* = 6), four patients underwent radical surgery followed by chemoradiotherapy; Case 12 received 33 fractions of PT and remained disease-free at 33-month follow-up. Two patients received postoperative radiotherapy alone: Case 14, who had concomitant primary hepatocellular carcinoma and poor performance status, received 33 fractions of radiotherapy and is currently undergoing ablative therapy for liver cancer; Case 15 received 30 fractions of Tomotherapy postoperatively and developed bone metastases at 6 months and lung metastases at 8 months. This patient is currently receiving second-cycle chemotherapy and immunotherapy. The remaining 5 patients were disease-free. Kaplan–Meier analysis was not performed for SMARCA4-deficient cases due to the very limited sample size and insufficient follow-up data.
- The SMARCA4-deficient TCS patient (Case 17) received 2 cycles of chemotherapy (paclitaxel and cyclophosphamide) plus 30 fractions of radiotherapy (dosage unknown) postoperatively and remained disease-free at 11 months of follow-up.

## 4. Discussion

SDSC represents a rare tumor subtype originating in the nasal cavity and paranasal sinuses. This aggressive phenotype stems from SWI/SNF complex subunit deficiencies, causing chromatin remodeling abnormalities and subsequent uncontrolled tumor proliferation. SMARCB1-deficient sinonasal carcinoma was first documented in 2014 [16] and subsequently recognized as a distinct subset of sinonasal undifferentiated carcinoma (SNUC) in the 2017 WHO Classification of Head and Neck Tumors (4th edition) [17]. These tumors remain extremely rare. However, SMARCA4-deficient sinonasal carcinoma is even rarer, with only a limited number of cases reported to date. SMARCA4-deficient sinonasal teratocarcinosarcoma (TCS) is also extremely rare and aggressive, and is characterized by intermixed teratomatous, carcinomatous, and sarcomatous elements. Its oncogenic mechanisms remain poorly understood. Rooper et al. [18] evaluated SMARCA4 staining in 22 TCS cases and reported loss of expression in 82% of cases. Consistent with previous reports indicating a male predominance across all three entities [19], our cohort had a notably higher male preponderance (82.4%). While SMARCA4-deficient tumors primarily arose in the nasal cavity, SMARCB1-deficient tumors demonstrated a predilection for the ethmoid sinus with or without nasal cavity extension, and frequently demonstrated skull base and orbital invasion in advanced stages. Clinical manifestations predominantly involved the nasal, ocular, and cranial regions. Although SDSC has only been formally recognized in the past decade with a limited number of reported cases, it accounts for 6% of primary sinonasal tumors and thus requires increased awareness among clinicians and pathologists [20].

Accurate diagnosis is a prerequisite for optimizing therapeutic strategies based on tumor morphology and molecular profile. The radiologic features observed in our cohort, including heterogeneous enhancement and aggressive bone destruction, may correlate with underlying histopathological characteristics such as tumor necrosis, high proliferative activity, and loss of differentiation. Together, these imaging–pathology correlations may represent potential diagnostic hallmarks of SDSC and assist in early recognition. The diagnosis of SDSC mainly relies on the loss of SMARCB1 or SMARCA4 expression in IHC. Under-recognition of these tumors may reflect both their rarity in the literature and the lack of routine SMARCB1/SMARCA4 IHC testing in some institutions. However, the histological features of SDSC vary significantly, with basaloid and plasmacytoid/rhabdoid morphologies being most common, followed by squamous, squamous papillary, glandular, clear-cell, and yolk-sac tumor differentiation [21,22]. Moreover, the heterogeneous morphology of TCS frequently results in diagnostic overlap with various other neoplasms. Rooper et al. [18] proposed that TCS and SMARCA4-deficient sinonasal carcinoma may represent a spectrum of related entities. Thus, SMARCA4 IHC may serve as a useful adjunct for rapid diagnosis of this challenging and perplexing entity. Additional IHC markers, including cytokeratin, synaptophysin, chromogranin, and SALL4, together with ancillary molecular studies such as Epstein–Barr virus-encoded RNA in situ hybridization and selected translocation analyses, may assist in the differential diagnosis of these histologically heterogeneous tumors [23]. High Ki-67 indices were observed across all three subtypes, suggesting increased proliferative activity. In our cohort, SMARCA4-deficient tumors appeared to have higher Ki-67 indices than SMARCB1-deficient carcinomas. However, due to the small sample size and the substantial proportion of missing Ki-67 and outcome data, no definitive association between Ki-67 expression and clinical outcomes could be identified, and this limitation should be taken into account when interpreting these findings. Although both SMARCB1 and SMARCA4 are key components of the SWI/SNF complex, they have distinct functional roles. SMARCB1 loss may allow partial preservation of catalytic activity, whereas SMARCA4 loss, involving a catalytic ATPase subunit, may lead to more profound impairment of chromatin remodeling, potentially accounting for differences in proliferation, clinical presentation, and tumor behavior.

To date, no specific treatments exist for these rare and highly aggressive diseases. The lack of comparative studies limits definitive conclusions, underscoring the need for further research. Currently, the treatment of SDSC is similar to that of other sinonasal skull base tumors, comprising surgical resection and adjuvant therapies such as radiotherapy and chemotherapy postoperatively [4]. Retrospective studies have investigated the response to induction chemotherapy guided by docetaxel, cisplatin and etoposide, which has roughly established the treatment paradigm for SNUC [24]. In SMARCA4-deficient lung cancer, platinum-based chemotherapy has shown efficacy [25]. Emerging therapeutic strategies for SWI/SNF-deficient malignancies include Enhancer of Zeste Homolog 2 (EZH2) inhibitors, CDK4/6 inhibitors, and immune checkpoint inhibitors [26,27,28,29,30,31,32]. However, their role in SDSC remains largely speculative and is not directly supported by data from the present cohort. Emerging evidence suggests that SWI/SNF deficiency may influence the tumor microenvironment by altering inflammatory signaling, antigen presentation, and immune-related transcriptional programs, potentially contributing to sensitivity to immune checkpoint inhibitors in a subset of tumors [33]. However, this effect appears to be heterogeneous across tumor types and molecular subgroups. In our cohort, five patients received immunotherapy as part of their treatment, including one as salvage therapy after brain metastasis (Case 1). Among SMARCB1-deficient cases, two patients (Cases 5 and 9) received chemotherapy combined with immunotherapy after biopsy, while one patient (Case 10) received immunotherapy but had unavailable follow-up data. Only one SMARCA4-deficient patient received immunotherapy. Due to the small sample size, heterogeneous treatment strategies, and incomplete follow-up information, a meaningful comparison between patients who received immunotherapy and those who did not was not feasible. In addition, PD-L1 expression, a potential predictive biomarker for response to immune checkpoint inhibitors, was not systematically available in our cohort. As PD-L1 testing was not routinely performed and data were incomplete for a substantial proportion of patients, its association with immunotherapy outcomes could not be assessed and warrants further exploration in subsequent studies.

In terms of radiotherapy, Case 6 only received PT after surgery. Regular follow-up examinations over 17 months showed no tumor recurrence or metastasis, experiencing only minor nasal dryness as a long-term effect. This indicates that PT may provide adequate target coverage while better sparing adjacent normal tissues, which is particularly relevant for treating childhood cancers, head and neck tumors, and brain tumors [34]. If patients receive charged particle therapy, such as proton or carbon ion therapy, higher radiation doses may be considered to achieve a higher relative biological effectiveness [21]. The efficacy of Proton Beam Therapy (PBT) was investigated by Russo, A.L. et al. [35] in a 17-year cohort of 54 sinonasal squamous cell carcinoma patients, and by Pasalic, D. et al. [36] in a cohort of 64 sinonasal carcinoma patients (including 14 SNUCs). Both studies confirmed that PBT provides excellent local control and overall survival with a favorable safety profile, lending support to the potential of achieving long-term control in SDSC.

With multimodal treatment, a substantial proportion of patients in our cohort achieved disease control. However, due to the insidious onset and lack of specific symptoms, the diagnosis of SDSC is usually delayed at advanced stages [37]. Notably, Case 15 developed bone and lung metastases shortly after radical surgery and radiotherapy, underscoring the aggressive nature and heterogeneous treatment response of SMARCA4-deficient tumors. Contributing factors may include high proliferative activity, early dissemination, delayed presentation due to mild initial symptoms, difficulty in achieving true oncologic margins in anatomically complex regions, and resistance to conventional therapies. Our findings are broadly consistent with previous reports showing poor outcomes in SDSC, including the pooled analysis by Lee et al. [22] and studies by Bishop [20]. However, interpretation should be cautious given the retrospective design, limited sample size, referral bias, heterogeneous treatment strategies, and variable follow-up duration. For patients with SMARCA4-deficient sinonasal carcinoma, the survival rate was 100% at last follow-up, although one patient developed metastases and remains on therapy. Favorable outcomes in some patients may have been related to complete surgical resection and timely postoperative therapy; however, causal relationships cannot be established in this retrospective study. Similarly, the favorable outcome in the patient with SMARCA4-deficient sinonasal TCS may have been associated with early-stage diagnosis and prompt multimodal treatment. A longer follow-up is needed to validate these observations. Case 4 highlights the potential for diagnostic confusion when metastatic lesions precede recognition of the primary sinonasal tumor. In Case 14, the coexistence of hepatocellular carcinoma may represent an unrelated comorbidity, although a paraneoplastic phenomenon, shared susceptibility, or treatment-related factors cannot be excluded. Despite these limitations, our study provides additional insights by integrating multiple SDSC subtypes within a single cohort, highlighting diagnostic challenges and offering real-world evidence on multimodal treatment approaches, including IT and PT. In the future, multimodal and multidisciplinary management of such refractory malignant tumors will remain the cornerstone of treatment.

## 5. Limitations

This study has several limitations. First, the small sample size, relatively short follow-up duration, and high censoring rate limited the precision of survival analysis; hence, median overall survival could not be reliably estimated, and Kaplan–Meier results should be interpreted with caution. We therefore focused primarily on 1- and 3-year DFS rates. Second, the retrospective design and inclusion of externally referred patients resulted in incomplete clinical data, particularly regarding surgical approaches, detailed treatment processes, treatment-selection rationale, Ki-67 indices, and PD-L1 expression, which restricted further analyses and may have introduced selection and information bias. Third, referral bias may have led to a higher proportion of complex or advanced cases, potentially limiting the generalizability of our findings. Finally, the heterogeneity of treatment strategies makes it difficult to distinguish the effects of specific therapies from the intrinsic biological behavior of the tumors. These factors should be taken into consideration when interpreting the results.

## 6. Conclusions

SDSC is a rapidly progressive sinonasal malignancy that frequently presents at an advanced stage. Our cohort expands the clinicopathological spectrum of these rare entities, highlighting subtype-specific differences and diagnostic challenges. Routine SMARCB1 and SMARCA4 IHC is recommended to improve diagnostic accuracy. Early radical surgery combined with multimodal therapy may contribute to disease control. Further prospective studies are needed to optimize management strategies.

## Figures and Tables

**Table 1:** Clinicopathologic features of patients with SDSC.

No.	Age(y)/Sex	Location	Presentation	Initial Stage	Primary Treatment	MTS (Months)	Outcome	Follow-Up (Months)	INI1/BRG1 ^†^	Ki-67
1	47/F	Left sphenoid sinus, ethmoid sinus, anterior cranial base	Epistaxis, nasal obstruction	T4aN0M0	Surgery + RT + CT	Lung (4), bone (35), brain (41)	DOD	57	INI1(−)	NA
2	43/M	Right ethmoid sinus, orbital contents	Visual impairment, monocular swelling and pain	T4aN0M0	Surgery + RT + CT	-	NED	54	INI1(−)	>30%
3	46/M	NA	Hyposmia, headache, dizziness	NA	Surgery + RT + CT	-	NED	36	INI1(−)	NA
4	58/F	Left maxillary sinus, ethmoid sinus, gingiva	Gingival pain, gingival sinus tract	T3N2cM1	Surgery + CT + RT	Meninges (NA), bilateral parapharyngeal lymph nodes (NA)	DOD	35	INI1(−)	NA
5	48/M	Right maxillary sinus, orbital contents	Epistaxis, facial pain	T4aN0M0	Biopsy + CT + IT	Lung (12), mediastinal lymph nodes (12), liver (15)	DOD	19	INI1(−)	50%
6	25/M	Right ethmoid sinus, anterior cranial base	Epistaxis, hyposmia, purulent nasal discharge, headache, dizziness, visual impairment, monocular swelling and pain	T4aN0M0	Surgery + PT	-	NED	17	INI1(−)	NA
7	44/M	Right nasal cavity, paranasal sinuses, anterior cranial base	Epistaxis, nasal obstruction, monocular swelling and pain	T4aN0M0	Surgery (non-radical) + CT + RT	-	AWD	12	INI1(−)	20%
8	36/M	Left ethmoid sinus	Epistaxis, nasal obstruction, headache	T3N0M0	Surgery + CT + RT	-	NED	10	INI1(−)	70%
9	55/F	NA	Epistaxis, nasal obstruction, visual impairment	NA	Biopsy + CT + IT	NA	NA	NA	INI1(−)	1%
10	60/M	Left ethmoid sinus, nasal cavity	Epistaxis, nasal obstruction, purulent nasal discharge	T2N0M0	Surgery + RT + CT + IT	Liver (12)	NA	NA	INI1(−-)	50%
11	62/M	Right nasal cavity, paranasal sinuses	Epistaxis, hyposmia, nasal obstruction, purulent nasal discharge, diplopia, visual impairment	T4aN0M0	Surgery + RT + CT + IT	-	NED	34	BRG1(−)	>70%
12	51/M	Left nasal cavity, ethmoid sinus, anterior cranial base, orbital contents	Visual impairment	T4aN0M0	Surgery + CT + PT	-	NED	33	BRG1(−)	70%
13	34/M	NA	Nasal obstruction	NA	Surgery + CT + RT	-	NED	21	BRG1(−)	90%
14	69/M	Right nasal cavity, ethmoid sinus, anterior cranial base	Epistaxis, nasal obstruction	T4aN0M0	Surgery + RT	-	NED	16	BRG1(−)	70%
15	58/M	Left ethmoid sinus, anterior cranial base	Epistaxis, purulent nasal discharge	T4aN0M0	Surgery + RT	Bone (6), lung (8)	AWD	10	BRG1(−)	70%
16	27/M	NA	Nasal obstruction, purulent nasal discharge	NA	Surgery + CT + RT	-	NED	8	BRG1(−)	70%
17	38/M	Left nasal cavity, ethmoid sinus	Epistaxis, nasal obstruction, purulent nasal discharge	T2N0M0	Surgery + CT + RT	-	NED	11	BRG1 (focal loss) *	70%

AWD, alive with disease; CT, chemotherapy; DOD, died of disease; F, female; IT, immunotherapy; M, male; MTS, metastasis (time to metastasis is calculated from the date of initial treatment); NA, not available, indicating missing data due to incomplete records or unavailable information, including follow-up status, pathological data, or timing of metastasis; NED, no evidence of disease; PT, proton therapy; RT, radiotherapy. * BRG1 loss in stromal components. ^†^ INI1 refers to SMARCB1 and BRG1 refers to SMARCA4.

## Data Availability

The original contributions presented in this study are included in the article. Further inquiries can be directed to the corresponding authors.

## Abbreviations

The following abbreviations are used in this manuscript (listed in alphabetical order):

AWD	Alive with disease
CT	Computed tomography
DFS	Disease-free survival
DOD	Died of disease
EZH2	Enhancer of zeste homolog 2
F	Female
IHC	Immunohistochemistry
IT	Immunotherapy
LITT	Laser interstitial thermotherapy
M	Male
MRI	Magnetic resonance imaging
MTS	Metastasis
NA	Not available
NED	No evidence of disease
PBT	Proton beam therapy
PT	Proton therapy
RT	Radiotherapy
SDSC	Switch/sucrose nonfermentable-deficient sinonasal carcinomas
SNUC	Sinonasal undifferentiated carcinoma
SWI/SNF	Switch/sucrose nonfermentable
TCS	Teratocarcinosarcoma
